# Comparing mechanochemical endovenous ablation using Flebogrif with endovenous laser ablation in the treatment of primary great saphenous vein incompetence: protocol for a multicentre, open-label, non-inferiority, observer-blinded, randomised controlled trial (REBORN trial)

**DOI:** 10.1136/bmjopen-2024-087490

**Published:** 2024-08-07

**Authors:** Sharon Oud, Tamana Alozai, Michiel A Schreve, Michael C Mooij, Clarissa J van Vlijmen, Çağdaş Ünlü

**Affiliations:** 1 Surgery, Amsterdam UMC Locatie AMC, Amsterdam, Netherlands; 2 Surgery, Noordwest Ziekenhuisgroep, Alkmaar, Netherlands; 3 Surgery, Onze Lieve Vrouwe Gasthuis, Amsterdam, Netherlands; 4 Skin and Vein Clinic Oosterwal, Alkmaar, Netherlands

**Keywords:** Vascular surgery, Surgical dermatology, VASCULAR SURGERY

## Abstract

**Introduction:**

Endovenous laser ablation (EVLA) is associated with an excellent outcome in the treatment of great saphenous vein (GSV) incompetence. However, the use of thermal ablation requires tumescent anaesthesia and is associated with a risk of thermal damage. Mechanochemical endovenous ablation (MOCA) is a non-thermal ablation (NTA) alternative, which combines mechanical endothelial damage with the infusion of a sclerosant liquid or foam. Tumescent anaesthesia is not required. Preliminary experiences with MOCA using the Clarivein device show less intraprocedural and postprocedural pain and a faster clinical improvement compared with EVLA. Flebogrif (Balton, Poland) is a relatively new MOCA device. To determine the role of MOCA using Flebogrif, a well-designed, randomised controlled clinical trial of sufficient sample size and follow-up time is required. In this article, we provide the study protocol for the REBORN trial, aiming to demonstrate that MOCA using Flebogrif is not inferior to EVLA for the outcome of anatomical success in the treatment of GSV incompetence.

**Methods and analysis:**

This multicentre, open-label, non-inferiority, observer-blinded, randomised controlled trial randomises patients who are diagnosed with GSV incompetence and aged 18–80 years between Flebogrif and EVLA. 310 patients in 3 participating centres (Northwest Clinics Alkmaar, Skin and Vein Clinic Oosterwal Alkmaar and Red Cross Hospital Beverwijk) will be included. The primary outcome is anatomical success at 12 months. Secondary outcomes are intraprocedural pain, operation time, technical success, postprocedural pain, safety, anatomical success during other follow-up moments, complications, clinical success, aesthetic result, disease-specific quality of life, reinterventions, anterior accessory saphenous vein reflux and neovascularisation. Patients will be followed up at 1 week, 1, 6, 12, 24 and 60 month(s) after treatment.

**Ethics and dissemination:**

The institutional review board (Medical Ethical Review Committee of the Vrije Universiteit Medical Center) approved this study on 17 May 2021 under case number 2020.0740. Written informed consent is obtained by the coordinating investigator from all participants prior to study enrolment. After completion of the trial, the results will be submitted to an international scientific journal for peer-reviewed publication.

**Trial registration number:**

Overzicht van Medisch-wetenschappelijk Onderzoek in Nederland, NL-OMON25145, previously NL9527; Centrale Commissie Mensgebonden Onderzoek, NL74491.029.20.

**STRENGTHS AND LIMITATIONS OF THIS STUDY**

- This study is a well-powered randomised controlled trial comparing mechanochemical ablation using Flebogrif with endovenous laser ablation in the treatment of great saphenous vein incompetence.
- The treatment will be blinded to outcome assessors during follow-up.
- Patients will be followed up for 5 years.
- The non-inferiority design does not allow for a superiority comparison.

## Introduction

 Chronic venous insufficiency (CVI) of the lower limbs is a common disorder. The prevalence of superficial vein reflux is 21% in the adult population, which increases linearly with age.^1^ CVI has been associated with decreased general and disease-specific quality of life (QoL).^2^,^4^ CVI is generally caused by insufficiency of the great saphenous vein (GSV).^5^

Minimally invasive endothermal treatment, for example, endovenous laser ablation (EVLA) or radiofrequency ablation (RFA), has become the first line of treatment for superficial venous reflux. This treatment strategy has the advantage of avoiding general anaesthesia, shortening operation time and decreasing postoperative pain and morbidity when compared with open surgery.^6^,^10^ However, the use of endothermal ablation techniques is associated with thermal damage to superficial nerves, skin burn, prolonged pain and the administration of tumescent anaesthesia can also be painful.^11^

Newer treatments modalities, especially non-thermal ablation (NTA) techniques, have potential benefits, such as increased patient acceptability and decreased risk of nerve injury. Currently, there are three NTA techniques that are widely discussed. Ultrasound-guided foam sclerotherapy (UGFS) has been used for some decades and avoids the risk of nerve injury. However, it is not as effective as endothermal ablation. Boersma *et al* reported pooled anatomical success rates of 63.6% after UGFS vs 98.5% and 97.1% after EVLA and RFA, respectively. Short-term clinical success and patient-reported outcome measures were comparable.^12^ Additionally, there is a very rare but well-documented risk of stroke after UGFS.^13^ More recently, two alternative NTA modalities have emerged. The first is cyanoacrylate closure using the VenaSeal Closure System or the VenaBLOCK Venous Closure System. Both devices enable the endovenous delivery of cyanoacrylate tissue adhesive to the vein causing fibrosis. A second alternative is mechanochemical endovenous ablation (MOCA), which combines mechanical endothelial damage with the infusion of a sclerosant liquid or foam. An example is the Clarivein device (Vascular Insights, USA), which combines a rotating wire causing endothelial damage with the injection of a sclerosant solution. The Clarivein device is studied and compared with endovenous thermal techniques in several randomised controlled trials. In both the MARADONA trial and Venefit versus Clarivein trial, pain scores were significantly lower in patients treated with Clarivein.^14 15^ However, these significant differences were not found in other clinical studies.^16 17^ Although overall, anatomical success is higher in patients treated with thermal ablation, similar improvements in clinical scores and QoL scores were found in all studies.^14^,^17^ However, the Clarivein device did not lead to widespread use.

In recent years, a new MOCA device has entered the market; the Flebogrif device (Balton, Poland). The Flebogrif device consists of hooks at the end of the catheter that damage the intima of the vein. This endothelial damage is also combined with the simultaneous injection of a sclerosant foam. No published literature is available that compares treatment using Flebogrif with treatment using Clarivein. The anatomical success rate of Flebogrif ranges from 90% to 96% after a follow-up time of 12 months.^18^,^20^ Iłżecki *et al* reported an anatomical success rate of 92% after 24 months.^21^ Only one study compared Flebogrif with EVLA in a randomised controlled trial, with occlusion rates of 96% vs 98%, respectively.^20^

To determine the role of MOCA using Flebogrif, a well-designed, randomised controlled clinical trial of sufficient sample size and follow-up time is required. The current study has been designed to demonstrate that MOCA using Flebogrif is not inferior to EVLA for the outcome of anatomical success in the treatment of GSV incompetence.

## Methods and analysis

The protocol was drafted in accordance with the Standard Protocol Items: Recommendations for Interventional Trials statements. The trial is registered at ‘Overzicht van Medisch-wetenschappelijk Onderzoek in Nederland, NL-OMON25145’ and ‘Centrale Commissie Mensgebonden Onderzoek, NL74491.029.20’. The trial was previously registered under the reference number NL9527.

### Study design

The REBORN trial is a two-arm, multicentre, open-label, non-inferiority, observer-blinded, randomised controlled trial. Patients with GSV incompetence referred to the Northwest Clinics Alkmaar, Skin and Vein Clinic Oosterwal Alkmaar and Red Cross Hospital Beverwijk will be screened for eligibility by the vascular surgeon or phlebologist in the outpatient clinic. All eligible patients will receive oral information about the study, an information letter and a participant consent form from the vascular surgeon or phlebologist. After receiving written informed consent, the research coordinator (TA/SO) performs computer randomisation and informs the patients about the treatment group they are allocated to. The intervention group will receive treatment with MOCA using the Flebogrif device. The control group will receive treatment using EVLA. The procedures will be performed by dedicated vascular surgeons. These vascular surgeons are experienced in performing laser therapy treatment, as this is the current standard treatment for GSV incompetence in our practices. Before treating study participants, all vascular surgeons will undergo training and complete a minimum of five supervised Flebogrif treatments to overcome the initial learning curve.

**Figure 1:**
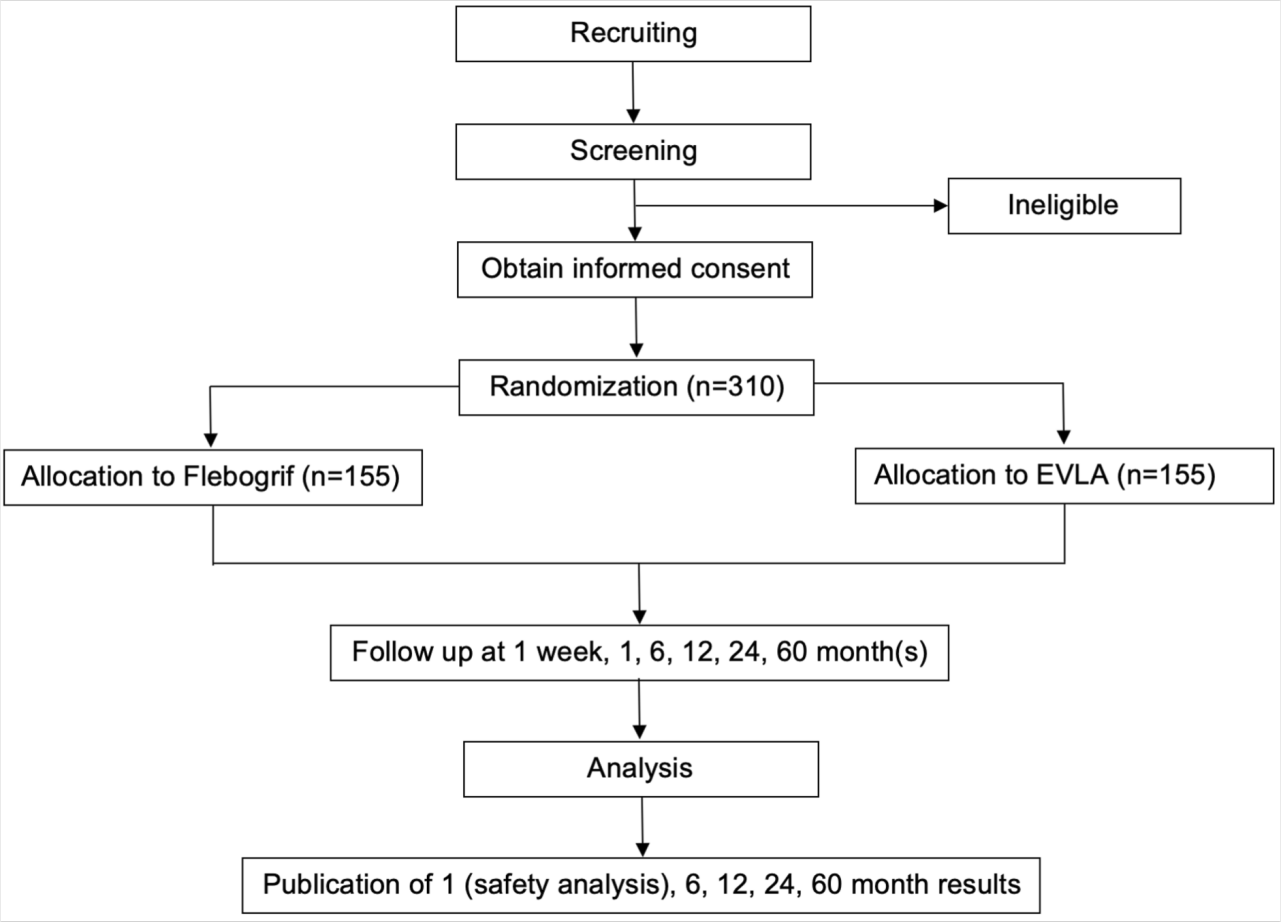
Study flow chart. EVLA, endovenous laser ablation.

Patients will be followed up at 1 week, 1, 6, 12, 24 and 60 month(s) after treatment. At each follow-up visit, patients will be seen at the outpatient clinic. A window of 4–14 days will be allowed for 1-week follow-up, 4–6 weeks for 1-month follow-up and ±1 month for 6, 12, 24 and 60 months follow-up. A study flow diagram is shown in figure 1, and table 1 shows all procedures and measurements performed at each follow-up visit.

**Table 1:** Study period, procedures and measurements

	Screening	Intervention	1 week	1 month	6 months	12 months	24 months	60 months
Informed consent	X							
Randomisation	X							
Flebogrif/EVLA		X						
Intraprocedural pain		X						
Operation time		X						
Technical success		X						
Postprocedural pain			X					
Safety				X				
DUS*	X	X	X	X	X	X	X	X
Complications		X	X	X	X	X	X	X
VCSS, CEAP	X		X	X	X	X	X	X
Aesthetic score	X		X	X	X	X	X	X
QoL questionnaire	X		X	X	X	X	X	X

* To assess the outcomes of anatomical success, anterior accessory saphenous vein () reflux and neovascularizsation.

CEAP, Clinical Etiology Anatomy Pathophysiology classification; DUS, duplex ultrasound; EVLA, endovenous laser ablation; QoL, quality of life; VCSS, Venous Clinical Severity Score

### Eligibility criteria

Patients are eligible for inclusion if they fulfil the following criteria:

- Age 18–80 years.
- Unilateral symptomatic primary GSV and saphenofemoral junction (SFJ) incompetence.
- GSV diameter ≥4 and ≤12 mm.
- Intrafascial GSV length ≥15 cm.

### Exclusion criteria

- Bilateral endovenous thermal/MOCA treatment of the GSV.
- Simultaneous ipsilateral endovenous thermal/MOCA treatment of additional veins.
- C6 varicose veins.
- Previous ipsilateral GSV or anterior accessory saphenous vein (AASV) treatment.
- Superficial thrombophlebitis or deep venous thrombosis (DVT) in the last 6 months.
- Occlusion of deep venous system.
- Coagulation disorders or increased risk of thromboembolism.
- Use of direct oral anticoagulants or vitamin K antagonists.
- Pregnancy or lactation.
- Immobilisation.
- Cognitive impairment or language barrier.
- Allergy or contraindication to polidocanol.
- Severe renal or liver insufficiency.

### Randomisation and blinding

Randomisation will be done by the coordinating investigator. Randomisation to one of two treatment groups will be on a 1:1 ratio. Randomisation will be performed in alternating blocks (4, 6, 8) and stratified on surgeon (n=7), using a web-based randomisation tool: Castor Electronic Data Capture (EDC). Due to the nature of the intervention, it is not possible to conceal the allocation group from either the patients or the treating surgeons. However, the treatment will be blinded to outcome assessors during follow-up as patients are told not to tell which treatment they received.

### Sample size calculation and drop-out

A sample size calculation is performed for the primary outcome of anatomical success at 12 months.

#### Anatomical success

The calculation is based on the hypothesis that MOCA using Flebogrif will have no inferior anatomical success 12 months after treatment, compared with EVLA. The anatomical success rate in the EVLA group is expected to be 95%.^22^,^25^ The anatomical success rate in the Flebogrif group is expected to be 93%.^18^,^20^ A non-inferiority margin of −10% is applied. This margin is based on the anatomical success rate of 85% after MOCA using Clarivein.^14 25 26^ Taking into account a power of 80% and a significance level of 2.5% (one-sided test on the lower level of the 95% CI of the expected difference), 278 patients will be needed (https://www.sealedenvelope.com/power/binary-noninferior/). Corrected for 10% lost to follow-up, 155 patients need to be included in each treatment group. The total study population will consist of 310 patients.

#### Withdrawal

Patients can leave the study at any time for any reason if they wish to do so without any consequences. The coordinating investigator can decide to withdraw a patient from the study for urgent medical reasons. After withdrawal, individual patients will not be replaced. If a patient is withdrawn from the study, follow-up ends.

### Study treatment

#### Intervention (Flebogrif) group

The included patients randomised to the intervention group will receive treatment with Flebogrif. The Flebogrif device is used to induce a combination of mechanical and chemical damage to the vein wall. The Flebogrif device is equipped with five retractable cutting/scratching elements that cause mechanical damage. The simultaneous, controlled administration of a foam sclerosant, results in chemical damage and obliteration of superficial venous trunks. No tumescent anaesthesia, sedation or antibiotics are required. The surgeons are experienced in the performance of endovascular procedures within the superficial venous system, with particular consideration of the specific nature and methodology of mechanochemical procedures. Prior to treatment, the GSV is marked using ultrasound. The patient is placed in supine position. Standard agents for disinfecting the operating field are used for skin desinfection. Thereafter, the leg is covered with sterile surgical drapes. Venipuncture is performed under ultrasound guidance with a 18G needle. A 0.035” guidewire is introduced through the 18G needle and placed at the level of the connection with the femoral vein (SFJ). The vascular sheath is introduced over the guidewire. After flushing the kit elements, the Flebogrif catheter, with a length of 60 cm, is inserted over the guidewire. The the working part of the tip is positioned at the predefined point, that is, 1 cm below the SFJ. Before releasing the cutting elements of the Flebogrif, the position of the catheter is checked. The cutting elements of the Flebogrif are fixated by clamping the clamping nut (2–3 cm per seconds). If the vein has a diameter of 8 mm or larger, two cutting passages will be performed over the entire length. The core part of the procedure consists of a steady withdrawal of the Flebogrif at a fixed speed, with concomitant injection of the sclerosant foam (3% polidocanol (EasyFoam), 2 mg/kg, 1 on 4 air). During foam injection, the groin is continuously compressed using the ultrasound head. The maximum amount of foam used will be 10 mL (50 cm). After the catheter is removed, manual pressure is applied to the area of the venipuncture. A detailed description of the Flebogrif design, enabling the correct use of the catheter as intended by the manufacturer, is contained in the operating instructions. After treatment, the deep venous system is checked using duplex ultrasound (DUS). If deemed necessary, additional phlebectomy of side branches is performed during the same session. A whole leg compression stocking (20–30 mm Hg) is applied continuously for the first 24 hours and continiued during the day for 1 week. Patients may resume normal activities immediately after the procedure but are given the advice to avoid peak pressure during the first week. If deemed necessary, additional sclerotherapy of side branches is performed during one of the follow-up visits. If additional sclerotherapy is performed, leg compression will be continued for 1 week.

#### Control (EVLA) group

The included patients randomised to the control group will receive treatment with EVLA. Prior to treatment, the GSV is marked using ultrasound. The patient is placed in supine position. Standard agents for disinfecting the operating field are used for skin desinfection. Thereafter, the leg is covered with sterile surgical drapes. Venipuncture is performed under ultrasound guidance with a 18G needle. A 6 F 11 cm sheet is introduced and followed by the introduction and positioning of the tip of the 1470 nm 2ring radial fibre (ELVeS Radial; Biolitec, Austria) 0.5 cm below the SFJ. Perivenous tumescent anaesthesia is administered using lidocaine 0.05% cold saline (5°C–10°C) and a roller pump (Nouvag Dispenser DP20). After administration of the tumescent fluid, the location of the tip of the laser fibre is checked. Laser treatment is performed using a continuous mode with a power of 10–12 watts. By slowly withdrawing the fibre, a targeted energy dose is delivered that is determined by the diameter of the GSV (approximately 80 J/cm). This process is repeated until the entire incompetent vein is ablated. After treatment, the deep venous system is checked using DUS. If deemed necessary, additional phlebectomy of side branches is performed during the same session. A whole leg compression stocking (20–30 mm Hg) is applied continuously for the first 24 hours and continued during the day for 1 week. Patients may resume normal activities immediately after the procedure but are given the advice to avoid peak pressure during the first week. If deemed necessary, additional sclerotherapy of side branches is performed during one of the follow-up visits. If additional sclerotherapy is performed, leg compression will be continued for 1 week.

### Primary outcome

#### Anatomical success (at 12 months)

Anatomical success (at 12 months) is objectified at 12 months follow-up using DUS. DUS is performed with the patient in the upright position, applying manual compression to the calf and using the Valsalva manoeuvre to detect flow and reflux. Occlusion is defined as a non-compressible vein (segment) that subsequently lacks flow and reflux. Recanalisation is defined as a compressible vein (segment) with flow or reflux.

Anatomical success outcomes (at 12 months) are defined as:

- Anatomical success: complete occlusion of the treated vein segment or a recanalised segment of ≤5 cm.
- Anatomical failure:
  - Partial recanalisation: a recanalised segment of >5 cm.
  - Complete recanalisation: recanalisation of the entire treated vein segment.

### Secondary outcomes

#### Intraprocedural pain

Intraprocedural pain is assessed during treatment using an 11-point Numeric Rating Scale (NRS) ranging from 0 (no pain) to 10 (worst pain imaginable).

#### Operation time

Time interval (minutes) between the venipuncture and removal of the catheter.

#### Technical success

The ability to position the device adequately, treat the vein as planned and occlude the treated vein directly after the procedure as proven by DUS.

#### Postprocedural pain

Postprocedural pain is assessed daily during the first week after treatment using an 11-point NRS ranging from 0 (no pain) to 10 (worst pain imaginable).

#### Safety

Occurrence of DVT, pulmonary embolism (PE) or nerve injury within 1 month after treatment.

#### Anatomical success (during other follow-up moments)

Anatomical success (during other follow-up moments) is objectified at 1 week, 1, 6, 24 and 60 month(s) follow-up using DUS. DUS is performed with the patient in the upright position, applying manual compression to the calf and using the Valsalva manoeuvre to detect flow and reflux. Occlusion is defined as a non-compressible vein (segment) that subsequently lacks flow and reflux. Recanalisation is defined as a compressible vein (segment) with flow or reflux.

Anatomical success outcomes (during other follow-up moments) are defined as:

- Anatomical success: complete occlusion of the treated vein segment or a recanalised segment of ≤5 cm
- Anatomical failure:
  - Partial recanalisation: a recanalised segment of >5 cm.
  - Complete recanalisation: recanalisation of the entire treated vein segment.

#### Complications

The occurrence of (thrombo)phlebitis, infection of the puncture wound, haematoma, hyperpigmentation, skin burn, DVT, PE, nerve injury, allergic reaction to polidocanol or migraine is assessed perioperatively and at 1 week, 1, 6, 12, 24 and 60 month(s) follow-up.

#### Clinical success

Venous Clinical Severity Score (VCSS) and/or Clinical Etiology Anatomy Pathophysiology classification (CEAP) Clinical score improvement of ≥1 point compared with baseline, assessed at 1 week, 1, 6, 12, 24 and 60 month(s) follow-up. Also, the overall changes in VCSS and CEAP Clinical score will be assessed during these follow-up moments.

#### Aesthetic result

Changes in aesthetic results are assessed using the aesthetic Numeric Analogue Scale ranging from 0 (unsatisfied) to 10 (very satisfied), at 1 week, 1, 6, 12, 24 and 60 month(s) follow-up and compared with baseline.

#### Disease-specific QoL

Changes in QoL are assessed using the VEINES-QOL/Sym (VEnous INsufficiency Epidemiological and Economic Study on Quality of Life/Symptoms) questionnaire,^27^ at 1 week, 1, 6, 12, 24 and 60 month(s) follow-up and compared with baseline.

#### Reinterventions

A reintervention is defined as a secondary treatment of the GSV in patients with anatomical failure after Flebogrif or EVLA treatment and assessed at 1 week, 1, 6, 12, 24 and 60 month(s) follow-up.

#### AASV reflux and neovascularisation

Ipsilateral reflux in the AASV and/or the occurrence of neovascularisation from the groin is assessed at 1, 6, 12, 24 and 60 month(s) follow-up.

### Other measurements

- Before treatment: gender (female or male); age; medical history (occurrence of migraine, asthma or chronic obstructive pulmonary disease, treatment of other leg vein(s)); use of platelet aggregate inhibitors; treatment side (right or left); baseline general pain score (assessed using an 11-point NRS ranging from 0 (no pain) to 10 (worst pain imaginable)); the presence of an (in)sufficient AASV and its confluence anatomy; GSV diameter (SFJ and mid-thigh) and length.
- During treatment: treated vein length; power in watts and total joules (EVLA); the amount of polidocanol foam used (Flebogrif); performance of concomitant phlebectomy of side branches.
- 1 week, 1, 6, 12, 24 and 60 month(s) follow-up: treatment of side branches (phlebectomy and/or sclerotherapy (foam vs liquid, amount and percentage of polidocanol used)).

### Data analysis plan

The intention-to-treat (ITT) population will include all patients randomised to one of the two treatment groups. These groups will be analysed according to the ITT principle (analysed as randomised). The per-protocol (PP) population will only include those patients who underwent the procedure they were randomised to. Statistical analysis will be performed by using SPSS (IBM SPSS Statistics V.25).

#### Primary outcome analysis

Anatomical success at 12 months of follow-up is a dichotomous variable and will be described as numbers with percentages. The difference between the two groups will be presented as a percentage with a 95% CI. If the lower limit of this CI does not exceed the non-inferiority margin of −10%, the intervention will be considered to be non-inferior. A second analysis will be performed in which data from the last follow-up visit before a reintervention will be used in patients undergoing a reintervention of the GSV after failure of primary treatment.

These analyses will be performed for both the ITT and the PP population. Patients with missing data on the primary outcome parameters will be excluded from analyses.

#### Secondary outcome analysis

Categorical variables (technical success, safety, anatomical success, complications, clinical success, reinterventions, AASV reflux and neovascularisation) will be described as numbers with percentages. Between-group differences will be analysed using the χ^2^ test or the Fisher’s exact test where appropriate.

Continuous variables that are single measurements (operation time, intraprocedural and postprocedural pain scores) will be described as means with an SD in the case of normally distributed data and as medians with an IQR in the case of a non-normal distribution. Between-group differences will be analysed using the independent samples t-test or the Mann-Whitney U test depending on the data distribution.

Continuous variables that are measured at several moments (VCSS, CEAP Clinical score, aesthetic result and VEINES-QOL/Sym) will be described as means with an SD in the case of normally distributed data and as medians with an IQR in the case of a non-normal distribution. Between-group differences and change over time will be analysed using a linear mixed model. This type of analysis handles missing data well, making imputation unnecessary.

#### Other outcome analysis

Categorical variables will be described as numbers with percentages. Between-group differences will be analysed using the χ^2^ test or the Fisher’s exact test where appropriate. Continuous variables will be described as means with an SD in the case of normally distributed data and as medians with an IQR in the case of a non-normal distribution. Between-group differences will be analysed using the independent samples t-test or the Mann-Whitney U test depending on the data distribution.

#### Interim analysis

A planned interim analysis will be performed on the safety endpoints when 25% of patients have been randomised and have completed the 1-month follow-up.

### Data collection and management

Data will be handled according to the EU General Data Protection Regulation and the Dutch Act on Implementation of the General Data Protection Regulation (in Dutch: Uitvoeringswet Algemene verordening gegevensbescherming). All data will be collected by the research coordinator using an electronic case report form in Castor EDC. The data will be entered into a database, where patients will be assigned a study number. Their personal information will not be entered into the database. A separate patient identification list will be kept by the research coordinator in case the study data need to be linked to individual patients. This list is only accessible to the study team. Members of the study team, the Dutch healthcare inspectorate and the monitor and auditor of the sponsor, will have access to the source data. All study data will be kept for 15 years.

### Adverse events and reportable events

All adverse events (AEs) reported spontaneously by the subject or observed by the research coordinator or other members of the study team will be recorded. The following events are considered reportable events:

- Any serious AE that has a causal relationship with the investigational device, the comparator or the investigation procedure or where such causal relationship is reasonably possible.
- Any device deficiency that might have led to a serious AE if appropriate action had not been taken, intervention had not occurred or circumstances had been less fortunate.
- Any new findings in relation to any event referred to in points (a) and (b).

The research coordinator will report all reportable events to the sponsor without undue delay but not later than 3 calendar days after obtaining knowledge of the event. Reportable events that are (possibly) related to the investigational procedure will be reported to the accredited Medical Ethical Review Committee (METc) that approved the protocol through the web portal ToetsingOnline using the form MDCG 2020-10/2. Reportable events that are not related to the investigational procedure will be reported to the manufacturer.

All AEs will be followed until they have abated, or until a stable situation has been reached. Depending on the event, follow-up may require additional tests or medical procedures as indicated, and/or referral to the general physician or a medical specialist. Serious AEs need to be reported till the end of the study within the Netherlands, as defined in the protocol.

### Monitoring and safety

This study will be monitored by a qualified Good Clinical Practice (GCP) monitor from the Northwest Academy. An initiation visit will be performed before the start of the study. Additionally, a monitor visit will be performed during the study (at least once a year) and a close-out visit will be performed at the end of the study. The monitor will have no competing interests. For this study, no data and safety monitoring board will be installed because the added risk of Flebogrif compared with standard EVLA is negligible.

### Patient and public involvement

None.

## Ethics and dissemination

### Ethics

The trial will be conducted in agreement with the current version of the Declaration of Helsinki, the GCP guidelines and in accordance with the Dutch Medical Research Involving Human Subjects Act. The METc of the Vrije Universiteit Medical Center approved of the study on 17 May 2021 under case number 2020.0740. Amendments are changes made to the research after a favourable opinion by the accredited METc has been given.

### Participant informed consent requirements

All eligible patients will receive oral information about the study, an information letter and an informed consent form from the vascular surgeon or phlebologist. Following a minimum reflection period of 1 week, verbal consent will be obtained by the vascular surgeon or phlebologist, and the research coordinator will contact the patients. The research coordinator will seek informed consent from those willing to participate, and participants will be asked to complete the participant consent form (online supplemental material) before randomisation.

### Protocol amendments

After METc approval, any significant protocol modifications will be communicated to relevant parties via email, and the register of the ‘Overzicht van Medisch-wetenschappelijk Onderzoek in Nederland’ and the ‘Centrale Commissie Mensgebonden Onderzoek’ will be promptly updated online.

#### Current study status

The first patient was enrolled on 24 September 2021. Based on the current inclusion rate, we anticipate completing participant enrolment around October 2024. The study will end 5 years after inclusion of the last patient.

### Dissemination

After completion of the trial, the results will be submitted to an international scientific journal for peer-reviewed publication.

## supplementary material

10.1136/bmjopen-2024-087490 online supplemental file 1
