# Supplementary material for: Comparing mechanochemical endovenous ablation using Flebogrif with endovenous laser ablation in the treatment of primary great saphenous vein incompetence: protocol for a multicentre, open-label, non-inferiority, observer-blinded, randomised controlled trial (REBORN trial)
Source: BMJ Open. 2024 Aug 7;14(8):e087490. doi: 10.1136/bmjopen-2024-087490 (PMC11404146; doi:10.1136/bmjopen-2024-087490)
Supplement: online supplemental file 1 [file bmjopen-14-8-s001.pdf]

SUPPLEMENTAL MATERIAL

Participant consent form (Dutch)

- Ik heb de informatiebrief gelezen. Ook kon ik vragen stellen. Mijn vragen zijn goed genoeg beantwoord. Ik had genoeg tijd om te beslissen of ik meedoe.
- Ik weet dat meedoen vrijwillig is. Ook weet ik dat ik op ieder moment kan beslissen om toch niet mee te doen met het onderzoek. Of om ermee te stoppen. Ik hoef dan niet te zeggen waarom ik wil stoppen.
- Ik weet dat ik op het moment van de behandeling niet zwanger mag zijn.
- Ik geef de onderzoeker toestemming om mijn huisarts/specialist die mij behandelt te laten weten dat ik meedoe aan dit onderzoek.
- Ik geef de onderzoeker toestemming om mijn huisarts/specialist informatie te geven over onverwachte bevindingen uit het onderzoek die van belang zijn voor mijn gezondheid.
- Ik geef de onderzoekers toestemming om mijn gegevens te verzamelen en gebruiken. De onderzoekers doen dit alleen om de onderzoeksvraag van dit onderzoek te beantwoorden. De gegevens zullen 15 jaar bewaard worden.
- Ik weet dat voor de controle van het onderzoek sommige mensen al mijn gegevens kunnen inzien. Die mensen staan in deze informatiebrief. Ik geef deze mensen toestemming om mijn gegevens in te zien voor deze controle.
- Wilt u in de tabel hieronder ja of nee aankruisen?

Ik geef toestemming om mijn gegevens 15 jaar te bewaren om dit te gebruiken voor ander onderzoek op het gebied van spataderen of de verdere ontwikkeling van de behandelmethode, zoals in de informatiebrief staat.	Ja ☐	Nee☐
Ik geef toestemming om mij eventueel na dit onderzoek te vragen of ik wil meedoen met een vervolgonderzoek, zoals in de informatiebrief staat.	Ja ☐	Nee☐

- Ik wil meedoen aan dit onderzoek.

Mijn naam is (proefpersoon): …………………………………

Handtekening: ………………………   Datum : __ / __ / __

Ik verklaar dat ik deze proefpersoon volledig heb geïnformeerd over het genoemde onderzoek. Wordt er tijdens het onderzoek informatie bekend die die de toestemming van de proefpersoon kan beïnvloeden? Dan laat ik dit op tijd weten aan

deze proefpersoon.

Naam onderzoeker (of diens vertegenwoordiger):……………………………….

Handtekening:……………………… Datum: __ / __ / __

De proefpersoon krijgt een volledige informatiebrief mee, samen met een getekende versie van het toestemmingsformulier.
