# Supplementary material for: Comparing mechanochemical endovenous ablation using Flebogrif with endovenous laser ablation in the treatment of primary great saphenous vein incompetence: protocol for a multicentre, open-label, non-inferiority, observer-blinded, randomised controlled trial (REBORN trial)
Source: BMJ Open. 2024 Aug 7;14(8):e087490. doi: 10.1136/bmjopen-2024-087490 (PMC11404146; doi:10.1136/bmjopen-2024-087490)
Supplement: Supplementary file 2 [file bmjopen-2024-087490.reviewer_comments.pdf]

PEER REVIEW HISTORY

BMJ Open publishes all reviews undertaken for accepted manuscripts. Reviewers are asked to complete a checklist review form and are provided with free text boxes to elaborate on their assessment. These free text comments are reproduced below.

ARTICLE DETAILS

**TITLE (PROVISIONAL)**	Comparing mechanochemical endovenous ablation using Flebogrif with endovenous laser ablation in the treatment of primary great saphenous vein incompetence: protocol for a multicenter, open-label, non-inferiority, observer-blinded, randomized controlled trial (REBORN trial)
**AUTHORS**	Oud, Sharon; Alozai, Tamana; Schreve, Michiel; Mooij, Michael; van Vlijmen, Clarissa; Ünlü, Çağdaş

VERSION 1 - REVIEW

**REVIEWER NAME**	***Davies, AH***
**REVIEWER AFFILIATION**	Imperial College London, Vascular
**REVIEWER CONFLICT OF INTEREST**	Nil
**DATE REVIEW RETURNED**	09-Jul-2023

**GENERAL COMMENTS**	1. Some key references missing.<br><br>2. Interesting primary outcome - 2 patency and post op pain. Think should be one at a defined time point.<br><br>3. No discussion about adjuvant treatments.<br><br>4. Why use EVLA as opposed to RFA?<br><br>5. No mention of other NTNT techniques.<br><br>6. Outcome section needs improving and presentation in order.<br><br>7. No mention of learning curve with techniques and competence of operators.<br><br>8. Interim analysis section - is blank.<br><br>9. No mention of reintervention rates.

**REVIEWER NAME**	***van den Bos, Renate R.***
**REVIEWER AFFILIATION**	Erasmus MC
**REVIEWER CONFLICT OF INTEREST**	NA
**DATE REVIEW RETURNED**	20-Sep-2023

**GENERAL COMMENTS**	Methods: your expected anatomical success is 95%, I believe that is slightly too positive (as you also reported in your own review already published). I would stick too a somewhat lower success rate.<br><br>I would advise to have only 1 primary outcome: anatomical success rate after 1 year. Intraprocedural pain should move to secondary outcomes and then you do not need a power calculation for that.<br><br>It is unclear whether you note presence of tributaries, I would do so. Because large tributaries could influence recurrence of reflux in the GSV, they can influence postprocedural pain if not treated concomitantly etc. And I wonder why concomitant tributaries are not treated at the same session with phlebectomies. First because it is patient-friendly and second if they are equally distributed in the two groups it should not influence the results.<br><br>Then your expected drop-out rate is really low, it is more realistic to take 10% drop-out rate.<br><br>I would also include recurrence after 1 year: ipsilateral refluxing SFJ, AASV and tributaries as secondary outcome.

VERSION 1 – AUTHOR RESPONSE

<u>Reviewer 1</u>

1. Some key references missing.

Response: Could you please specify this question?

2. Interesting primary outcome - 2 patency and post op pain. Think should be one at a defined time point.

Response: We intentionally selected these two primary outcomes. Anatomical success at 12 months was chosen due to its common use in related studies, facilitating comparisons. Our hypothesis is that Flebogrif treatment's anatomical outcomes are non-inferior to laser therapy at 12 months. Our first power calculation is based on this hypothesis. Given the high success rates of laser therapy, we don't anticipate superior anatomical outcome with Flebogrif. Instead, we believe Flebogrif's benefits lie, in part, in the secondary outcome: intraprocedural pain. We conducted a power calculation for this primary outcome to ensure a sufficient participant number for potential significant differences. Our overall hypothesis is that Flebogrif treatment is less painful than laser therapy, with comparable anatomical results. If Flebogrif results in less pain but significantly worse anatomical outcome, the disadvantage may outweigh the advantage. The same consideration applies to the second primary outcome; if Flebogrif doesn't reduce pain, there may be no advantage to this relatively new therapy.

3. No discussion about adjuvant treatments.

Response: After one week, patients may undergo additional sclerotherapy if necessary. This is also a common practice in our current clinical setting. We understand that this may impact the results.

However, this will occur in both groups: both in the EVLA group and the Flebogrif group. Given that we apply additional sclerotherapy in both groups, we expect that these treatments are equally distributed in the two groups and that its impact on the results is kept as minimal as possible.

4. Why use EVLA as opposed to RFA?

Response: For feasibility reasons, literature reports comparable outcomes for EVLA compared to RFA. In the hospitals and clinics where this study is conducted, EVLA is the standard treatment modality for thermal ablation.

5. No mention of other NTNT techniques.

Response: We have mentioned the following NTNT techniques in the introduction: Clarivein (MOCA), ultrasound-guided foam sclerotherapy (UGFS), and cyanoacrylate glue occlusion (CAE). **See redline manuscript page 6 line 15-33.**

6. Outcome section needs improving and presentation in order.

Response: Thank you for these valuable comment. We have arranged all outcome variables, especially the secondary outcome measures, in the same order throughout the entire manuscript (abstract , methods, and analysis section). We have also maintained this order in Table 1 and made the necessary adjustments. We hope it is now more organized and clear.

7. No mention of learning curve with techniques and competence of operators.

Response: We already mentioned that the treatment will be done by vascular surgeons experienced in venous disease. **See redline manuscript page 8 line 19.** To make it more clear we added the following: These vascular surgeons are experienced in performing laser therapy treatment, as it is the current standard treatment for GSV incompetence in our practices. Before treating study participants, all vascular surgeons underwent training and completed a minimum of 5 supervised Flebogrif treatments to overcome the initial learning curve. **See redline manuscript page 8 line 19-23.**

8. Interim analysis section - is blank.

Response: We have explained when we will conduct an interim analysis based on the safety endpoint, and we have clarified what we include under this safety endpoint. **See redline manuscript page 18 line 9-11 and page 14 line 27-29.**

9. No mention of reintervention rates.

Response: Thank you for this valuable suggestion. We have added reinterventions as a secondary outcome measure. **See redline manuscript page 16 line 12-14.**

Reviewer 2

Methods: your expected anatomical success is 95%, I believe that is slightly too positive (as you also reported in your own review already published). I would stick too a somewhat lower success rate.

Response: Our power calculation is based on the study by Tawfik et al., which, at the time of drafting the protocol, stood out as the most relevant and comparable study to ours. This trial was the sole randomized study comparing Flebogrif to EVLA, whereas the other studies (included in the review) were prospective case studies and a prospective comparative study. Additionally, reducing our expected anatomical success rate for Flebogrif treatment would significantly alter our power calculation. This would require a much larger group of participants, resulting in a significant prolongation of the study duration, of which the practical implementation is not feasible and undesirable.

I would advise to have only 1 primary outcome: anatomical success rate after 1 year. Intraprocedural pain should move to secondary outcomes and then you do not need a power calculation for that.

Response: See our response to comment 2 from reviewer 1.

It is unclear whether you note presence of tributaries, I would do so. Because large tributaries could influence recurrence of reflux in the GSV, they can influence postprocedural pain if not treated concomitantly etc. And I wonder why concomitant tributaries are not treated at the same session with phlebectomies. First because it is patient-friendly and second if they are equally distributed in the two groups it should not influence the results.

Response: We do not specifically document the presence of tributaries. However, if deemed necessary by the surgeon and/or phlebologist, (large) tributaries are treated with phlebectomy during the same session or with (foam) sclerotherapy after 1 week. This data is being recorded and will be included in the results. (Foam) sclerotherapy was not administered during the same session due to the maximum dosage of foam that could be used, as it is already utilized during the Flebogrif treatment. We also added this to the methods section. **See redline manuscript page 13 line 10-11 and line 29-31.**

Then your expected drop-out rate is really low, it is more realistic to take 10% drop-out rate.

Response: Although some studies mention higher dropout rates, there are also studies with comparable dropout rates at 12 months follow-up (***ref. 1***). Additionally, increasing our expected dropout rate would significantly alter our power calculation. This would require a much larger group of participants, resulting in a significant prolongation of the study duration, of which the practical implementation is not feasible and undesirable.

Ref 1*: Randomized clinical trial comparing endovenous laser ablation of the great Saphenous vein with and without ligation of the sapheno-femoral junction: 2-year results. PMID: 18848791 DOI: 10.1016/j.ejvs.2008.08.015*

I would also include recurrence after 1 year: ipsilateral refluxing SFJ, AASV and tributaries as secondary outcome.

Response: We added ipsilateral reflux in the anterior accessory saphenous vein (AASV) and neovascularization from the groin as secondary outcome measures to the abstract, methods, and analysis section. **See redline manuscript page 16 line 16-18.**

VERSION 2 – REVIEW

**REVIEWER NAME**	***Davies, AH***
**REVIEWER AFFILIATION**	Imperial College London, Vascular
**REVIEWER CONFLICT OF INTEREST**	none
**DATE REVIEW RETURNED**	01-Dec-2023

**GENERAL COMMENTS**	1. Not convinced by 2 primary putcomes: one occlusion and the other periprocedural pain<br>2. ? Shoud be an equivalence trial rather than non inferiority<br>3. Occlusion rate is non inferiority, whereas pain would be a superiority study.<br>4.? QoL should be primary outcome as per CLASS trial<br>5. Not convinced re sample size calculations with respect to input data.<br>6. MOCA and RFA data available

**REVIEWER NAME**	***van den Bos, Renate R.***
**REVIEWER AFFILIATION**	Erasmus MC
**REVIEWER CONFLICT OF INTEREST**	NA
**DATE REVIEW RETURNED**	28-Nov-2023

**GENERAL COMMENTS**	Thank for your answers and adjustments.<br>There are some questions remaining. My question on power analysis and drop out: what you could consider is that you enlarge your non-inferiority interval to 10 percent and your drop-out rate to 10%. In that case you will not need more patients than your current power analysis. Otherwise, you will have a problem when dropout rate is higher than 5%, then you won't be able to make your point. Also, I still believe intraprocedural pain should be replaced to secondary outcomes, the argument that you did a power calculation is not applicable, you need much less patients than your primary outcome, and a secondary outcome can also be used as an argument for choosing a treatment if non-inferior in the primary outcome. (If you consider intraprocedural pain difference of 2 on a scale from 0-10 relevant at all..) Then you added esthetic outcome as a secondary outcome, but you can imagine that the esthetic outcome is heavily influenced by SVT's, treatment of side branches

	and remaining tributaries so another argument to note the presence and treatment of side-branches.

VERSION 2 – AUTHOR RESPONSE

Reviewer 1

1. Not convinced by 2 primary outcomes: one occlusion and the other periprocedural pain

Response: We adjusted the primary and secondary outcome(s). Anatomical success at 12 months is now the sole primary outcome. Intraprocedural pain is a secondary outcome for which we subsequently removed the sample size calculation.

2. ? Should be an equivalence trial rather than non inferiority

Response: Since anatomical success at 12 months is now the only primary outcome, this study is a non-inferiority trial. The hypothesis is as followed: treatment for great saphenous vein incompetence using Flebogrif is non-inferior to treatment using endovenous laser ablation for the outcome of anatomical success at 12 months (non-inferiority margin -10%).

3. Occlusion rate is non inferiority, whereas pain would be a superiority study

Response: Thank you for this valuable comment. Since intraprocedural pain is now a secondary outcome, this study is a non-inferiority trial (for the outcome of anatomical success at 12 months).

4. ? QoL should be primary outcome as per CLASS trial

Response: We chose the primary outcome of anatomical success at 12 months to be able to compare our main results to that of most other relevant studies assessing endovenous ablation techniques for the treatment of great saphenous vein incompetence. Since our sample size calculation is based on the outcome of anatomical success and we already included more than half of the patients, it is undesirable to change the primary outcome at this stage of the trial. However, we recognize the importance of QoL and if no other studies use this as their primary outcome, we won't be able to make comparisons in the future either.

If we were to perform a sample size calculation for the outcome of QoL, a 0.3 SD (medium effect size) with a two-sided alpha of 5% and 90% power can be detected in the VEINES-QoL/Sym questionnaire score if 285 patients are included. Correcting for 10% drop-out, 317 patients will be needed, which is almost equal to our sample size of 310 patients. When accepting a higher SD (0.5 for example), less patients will be needed and our sample size would be more than sufficient.

5. Not convinced re sample size calculations with respect to input data

Response: We adjusted our sample size calculation and updated the references used for this calculation. The expected anatomical success rate in the EVLA group remained 95%, but we supported this with the use of additional relevant studies. We lowered the expected anatomical success rate of Flebogrif from 95% to 93%, also based on additional relevant studies published before the start of our trial. In hindsight, the anatomical success rate of 93% can be supported by a

systematic review and meta-analysis of MOCA using Flebogrif published by Alozai et al. in 2022 (after the start of our trial).[1] The non-inferiority margin is enlarged from -7% to -10%. This is based on expert opinion within our study group and additional references on the anatomical success rate of MOCA using Clarivein. At last, we increased the drop-out rate from 5% to 10%, which is more realistic. These adjustments result in the need for 310, instead of 320, study patients.

See main document - marked manuscript page 10 line 25-33.

1. Alozai T, Huizing E, Schreve M, Mooij MC, van Vlijmen CJ, Wisselink W, Ünlü Ç. A systematic review and meta-analysis of mechanochemical endovenous ablation using Flebogrif for varicose veins. J Vasc Surg Venous Lymphat Disord. 2022 Jan;10(1):248-257.e2. doi: 10.1016/j.jvsv.2021.05.010. Epub 2021 Jun 6. PMID: 34091106.

6. MOCA and RFA data available

Response: There are several randomized controlled/clinical trials comparing MOCA using Clarivein with RFA.[2, 3] Lane et al. assessed periprocedural pain secondary to truncal ablation between MOCA and RFA. However, since pain is no longer a primary outcome, we removed the sample size calculation for this outcome. Holewijn et al. assessed postprocedural pain and anatomical success at 1- and 2-year follow up. At one year, the anatomical success rate was 84% for Clarivein and 94% for RFA. As mentioned before, we based the expected anatomical success rates of Flebogrif and EVLA on studies specifically assessing these two endovenous treatment modalities (Flebogrif and EVLA). However, we added this last reference to support the non-inferiority margin of -10%.

See main document - marked manuscript page 10 line 28-29.

There is one study comparing MOCA using Flebogrif with RFA[4]. However, this is a retrospective study with a follow-up of 6 weeks that was published after the start of our trial.

2. Lane T, Bootun R, Dharmarajah B, Lim CS, Najem M, Renton S, Sritharan K, Davies AH. A multi-centre randomised controlled trial comparing radiofrequency and mechanical occlusion chemically assisted ablation of varicose veins - Final results of the Venefit versus Clarivein for varicose veins trial. Phlebology. 2017 Mar;32(2):89-98. doi: 10.1177/0268355516651026. Epub 2016 Jul 9. PMID: 27221810.

3. Holewijn S, van Eekeren RRJP, Vahl A, de Vries JPPM, Reijnen MMPJ; MARADONA study group. Two-year results of a multicenter randomized controlled trial comparing Mechanochemical endovenous Ablation to RADiOfrequeNcy Ablation in the treatment of primary great saphenous vein incompetence (MARADONA trial). J Vasc Surg Venous Lymphat Disord. 2019 May;7(3):364-374. doi: 10.1016/j.jvsv.2018.12.014. PMID: 31000063.

4. Razaque A, Shazlee MK, Qamar S, Hyder SMS, Adel H. Early Results of Mechanochemical Ablation by Flebogrif and Radiofrequency Ablation in Treating Primary Varicose Veins of Lower Limb. Cureus. 2023 Sep 24;15(9):e45874. doi: 10.7759/cureus.45874. PMID: 37885503; PMCID: PMC10598325.

Reviewer 2

1. There are some questions remaining. My question on power analysis and drop out: what you could consider is that you enlarge your non-inferiority interval to 10 percent and your drop-out rate to 10%. In that case you will not need more patients than your current power analysis. Otherwise, you will have a problem when dropout rate is higher than 5%, then you won't be able to make your point.

Response: Thank you for this valuable comment. We increased our drop-out rate to 10% and enlarged the non-inferiority margin from -7% to -10%. We supported this with additional references on the anatomical success rates of MOCA using Clarivein.

See main document - marked manuscript page 10 line 28-29 and page 10 line 32-33.

2. Also, I still believe intraprocedural pain should be replaced to secondary outcomes, the argument that you did a power calculation is not applicable, you need much less patients than your primary outcome, and a secondary outcome can also be used as an argument for choosing a treatment if non-inferior in the primary outcome. (If you consider intraprocedural pain difference of 2 on a scale from 0-10 relevant at all..) Then you added esthetic outcome as a secondary outcome, but you can imagine that the esthetic outcome is heavily influenced by SVT's, treatment of side branches and remaining tributaries so another argument to note the presence and treatment of side-branches.

Response: Thank you again for this valuable comments. We adjusted the primary and secondary outcome(s). Anatomical success at 12 months is now the sole primary outcome. Intraprocedural pain is a secondary outcome for which we subsequently removed the sample size calculation.

Regarding mentioning the presence and treatment of side-branched: we do mention the treatment of side branches by either phlebectomy and/or (foam)-sclerotherapy. Side-branches are treated if deemed necessary by the treating physician and when desired by the patients. However, since we already included more than half of the patients, we are unable to retrospectively mention the presence of side-branched for all patients as this this parameter is not prospectively recorded. However, the nature of a randomized controlled trial increases the likelihood that the presence of side branches and their severity will be equally distributed across both treatment groups, therefore limiting the influence o

VERSION 3 – REVIEW

**REVIEWER NAME**	***Davies, AH***
**REVIEWER AFFILIATION**	Imperial College London, Vascular
**REVIEWER CONFLICT OF INTEREST**	Nil
**DATE REVIEW RETURNED**	27-Jun-2024

**GENERAL COMMENTS**	1. Key references of MOCA vs thermal missing<br>2. Referees only to Venaseal glue as NTNT, need to acknowledge Turkish glue

**REVIEWER NAME**	***van den Bos, Renate R.***
**REVIEWER AFFILIATION**	Erasmus MC
**REVIEWER CONFLICT OF INTEREST**	NA
**DATE REVIEW RETURNED**	10-Jul-2024

**GENERAL COMMENTS**	I am satisfied with the corrections made. Design is improved.

VERSION 3 – AUTHOR RESPONSE

Reviewer 1

7. Key references of MOCA vs thermal missing

Response: We added three relevant references comparing MOCA using Clarivein versus endovenous thermal ablation techniques to the introduction:

'The Clarivein device is studied and compared to endovenous thermal techniques in several randomized controlled trials. In both the MARADONA trial and Venefit™ versus Clarivein® trial, pain scores were significantly lower in patients treated with Clarivein.[14,15] However, these significant differences were not found in other clinical studies.[16,17] Although overall, anatomical success is higher in patients treated with thermal ablation, similar improvements in clinical scores and QoL scores were found in all studies.[14-17]'

15. Lane, T. *et al.* A multi-centre randomised controlled trial comparing radiofrequency and mechanical occlusion chemically assisted ablation of varicose veins – Final results of the Venefit versus Clarivein for varicose veins trial. *Phlebology* **32**, 89–98 (2017).

16. Vähäaho, S. *et al.* Randomized clinical trial of mechanochemical and endovenous thermal ablation of great saphenous varicose veins. *Br. J. Surg.* **106**, 548–554 (2019).

17. Mohamed, A. H. *et al.* A Randomized Controlled Trial of Endovenous Laser Ablation Versus Mechanochemical Ablation with ClariVein in the Management of Superficial Venous Incompetence (LAMA Trial). *Ann. Surg.* **273**, E188–E195 (2021).

See main document - marked manuscript page 5 line 28-33.

8. Referees only to Venaseal glue as NTNT, need to acknowledge Turkish glue

Response: Thank you for this comment, we added the 'VenaBLOCK® Venous Closure System' to the text.

See main document - marked manuscript page 5 line 23-24.

<u>Reviewer 2</u>

I am satisfied with the corrections made. Design is improved.

Response: Thank you for this comment.
